# Supplementary material for: Modelling seasonality of Lassa fever incidences and vector dynamics in Nigeria
Source: PLoS Negl Trop Dis. 2023 Nov 13;17(11):e0011543. doi: 10.1371/journal.pntd.0011543 (PMC10681316; doi:10.1371/journal.pntd.0011543)
Supplement: S1 Appendix — (PDF) [file pntd.0011543.s001.pdf]

Supplementary Appendix

James Q. McKendrick*, Warren S. D. Tennant, Michael J. Tildesley

* j.mckendrick@warwick.ac.uk

Contents

1 Mathematical analysis **2**
1.1 Disease-Free Periodic Solution 2
1.2 Vector endemic equilibrium and stability 3
1.3 Reproduction ratio 4

2 Results **5**

3 Sensitivity Analysis **6**

Github repository with model, fitting code and results available at https://github.com/JimmyMckendrick1/LassaFever_SeasonalityNigeria

1 Mathematical analysis

1.1 Disease-Free Periodic Solution

In this section, we study the existence and uniqueness of the disease-free periodic solution of system defined in equation set (3) of **Materials and methods**.

We define our initial condition $\mathcal{A}_{\prime} = (S_r(0), I_r(0), R_r(0), S_h(0), E_h(0), A_h(0), I_h(0), R_h(0)) \in \mathcal{R}^8_+$. In the case of the disease-free solution we have $(S_r(0), 0, 0, S_h(0), 0, 0, 0, 0)$. We therefore only need consider the differential equations for S_r and S_h.

$$\begin{aligned}\frac{\mathrm{d}S_r}{\mathrm{d}t} &= B(t)N_r - \frac{\beta_{rr}S_rI_r}{N_r} - \mu_r S_r \\ &= (B(t) - \mu_r)S_r\end{aligned}$$

Since $N_r = S_r$ and $I_r = 0$. Hence

$$S_r(t) = S_r(0)e^{(\int_0^t B(\tau)-\mu\,\mathrm{d}\tau)}$$

where

$$B(t) = K\exp\left\{-s\cos\left(\pi(\frac{t}{365} - \phi)\right)^2\right\} \quad \text{and } K = \frac{\pi\mu_r e^{s/2}}{\int_0^\pi e^{-s/2\cos(x)}\,\mathrm{d}x}$$

Proceeding and ignoring the time translation ϕ, since

$$\cos\left(\frac{\pi t}{365}\right)^2 = \frac{1}{2}(\cos\left(\frac{2\pi t}{365}\right)) + 1)$$

With the substitution $t' = \frac{\pi t}{365}$,

$$\begin{aligned}\int_0^{365} B(t)\,\mathrm{d}t &= \frac{365K}{\pi}\int_0^\pi \exp\Big(-\frac{s}{2}\cos(2t')\Big)e^{-s/2}\,\mathrm{d}t' = \frac{365Ke^{-s/2}}{\pi}\int_0^\pi \exp\Big(-\frac{s}{2}\cos(x)\Big)\,\mathrm{d}x \\ &= 365\mu_r = \int_0^{365}\mu_r dt\end{aligned}$$

Thus the population size is periodic year-on-year, as per Peel et al (2014), hence $S_r(t)$ is periodic [1].

Solving for S_h:

$$\begin{aligned}\frac{\mathrm{d}S_h}{\mathrm{d}t} &= B_hN_h - (\beta_{rh}I_r + \beta_{hh}(A_h + I_h))\frac{S_h}{N_h} - \mu_h S_h \\ &= (B_h - \mu_h)S_h\end{aligned}$$

Since $N_h = S_h$ and $I_r, A_h, I_h = 0$. Therefore $S_h(t) = S_h(0)e^{(B_h - \mu_h)t}$.

1.2 Vector endemic equilibrium and stability

Suppose $(S_r(0), I_r(0), R_r(0)) \in \mathcal{R}^3_+$ is a non-trivial and all positive solution to the system of equations (3) in section 2.1. By setting the LHS of equation set (3) to be 0 then we obtain the following:

$$\frac{\mathrm{d}I_r}{\mathrm{d}t} = \frac{\beta_{rr} S_r^* I_r^*}{N_r^*} - (\gamma_r + \mu_r) I_r^* = 0$$
$$\implies S_r^* = \frac{\gamma_r + \mu_r}{\beta_{rr}} N_r^*$$

Which exists and is unique from the disease free equilibrium if and only $\beta_{rr} > \gamma_r + \mu_r$.

$$I_r^* = N_r\left(\frac{B(t)}{\mu_r + \gamma_r} - \frac{\mu_r}{\beta_{rr}}\right)$$

Which exists and is unique from the disease free equilibrium all $t > 0$ if and only $B(t) > \frac{\mu_r(\mu_r+\gamma_r)}{\beta_{rr}}$.

For ease of calculations we now consider the relative proportions of the population with $\hat{S}_r = \frac{S_r}{N_r}$ and $\hat{I}_r = \frac{I_r}{N_r}$ and let $\mathcal{R} = \frac{\beta_{rr}}{\gamma_r+\mu_r}$. The Jacobian, $\hat{J}$, of the normalised system for $(\hat{S}_r, \hat{I}_r)$ is

$$\hat{J}(\hat{S}, \hat{I}) = \begin{bmatrix} -\beta_{rr}\hat{I} - \mu_r & -\beta_{rr}\hat{S} \\ \beta_{rr}\hat{I} & \beta_{rr}\hat{S} - (\gamma_r - \mu_r) \end{bmatrix}$$

and at the state of endemic equilibrium $(\hat{S_r^*}, \hat{I_r^*}) = (\frac{1}{\mathcal{R}}, \frac{B(t)}{\mu_r+\gamma_r} - \frac{\mu_r}{\beta_{rr}})$

$$\hat{J}(\hat{S^*}, \hat{I^*}) = \begin{bmatrix} -\mathcal{R}B(t) & -(\gamma_r + \mu_r) \\ \mathcal{R}B(t) - \mu_r & 0 \end{bmatrix}$$

thus giving the following characteristic equation

$$\lambda^2 + \mathcal{R}B(t)\lambda + (\mathcal{R}B(t) - \mu_r)(\gamma_r + \mu_r) = 0,$$

which has the following eigenvalues:

$$\lambda_\pm = \frac{-B(t)\mathcal{R} \pm \sqrt{B(t)^2\mathcal{R}^2 - 4(\mathcal{R}B(t) - \mu_r)(\gamma_r + \mu_r)}}{2}.$$

Since $B(t)$ is a time-dependent function it may become negligible or more dominant in the above equation. In the case that $B(t)$ is very large such that $\mathcal{R}B(t) \gg \gamma_r + \mu_r > \mu_r$ we see that the eigenvalues are approximately

$$\begin{aligned} \lambda_+ &= \frac{-B(t)\mathcal{R} + \sqrt{(B(t)\mathcal{R} - 2(\gamma_r + \mu_r))^2 - 4(\gamma + \mu_r)^2 + 4\mu_r(\gamma_r + \mu_r)}}{2} \\ &< \frac{-B(t)\mathcal{R} + \sqrt{B(t)^2\mathcal{R}^2 - 4(\gamma + \mu_r)^2 + 4\mu_r(\gamma_r + \mu_r)}}{2} \\ &\approx \frac{-B(t)\mathcal{R} + \sqrt{B(t)^2\mathcal{R}^2}}{2} \\ &= 0. \end{aligned}$$

Therefore the vector system has an endemic fixed point is stable when $\mathcal{R}B(t) \gg \gamma_r + \mu_r > \mu_r$ is met, which may vary over time and is subject to the parameters for transmission and recruitment of the vector.

1.3 Reproduction ratio

First, we observe that the compartments which indicate infected individuals of any description are I_r, E_h, A_h and I_h. Ordered as X_1, X_2, X_3, X_4 as was written we obtain the generation matrix, F, where the entry $F_{i,j}$ is equal to the rate of new infections in compartment i and partially differentiated with respect to X_j.

$$F(t) = \begin{bmatrix} \frac{\beta_{rr} S_r}{N_r} & 0 & 0 & 0 \\ \frac{\beta_{rh} S_h}{N_h} & 0 & \frac{\beta_{hh} S_h}{N_h} & \frac{\beta_{hh} S_h}{N_h} \\ 0 & 0 & 0 & 0 \\ 0 & 0 & 0 & 0 \end{bmatrix}$$

Further more we can construct the matrix V representing transfer of individuals from each compartment by means other than infection transmission, with $V_{i,j} = V^-_{i,j} - V^+_{i,j}$ and $V^-_{i,j}$ being the rate of individuals out of the compartment partially differentiated by X_j and the entries in $V^+_{i,j}$ being the rate coming into that compartment. We constructed it as follows:

$$V(t) = \begin{bmatrix} (\gamma_r + \mu_r) & 0 & 0 & 0 \\ 0 & (\mu_h + \nu) & 0 & 0 \\ 0 & -p\nu & \mu_h + \gamma_h & 0 \\ 0 & -(1-p)\nu & 0 & (\mu_h + \mu_{h_I} + \gamma_h) \end{bmatrix}$$

therefore

$$V^{-1}(t) = \begin{bmatrix} \frac{1}{(\gamma_r+\mu_r)} & 0 & 0 & 0 \\ 0 & \frac{1}{(\mu_h+\nu)} & 0 & 0 \\ 0 & \frac{p\nu}{(\mu_h+\gamma_h)(\mu_h+\nu)} & \frac{1}{\mu_h+\gamma_h} & 0 \\ 0 & \frac{(1-p)\nu}{(\mu_h+\nu)\left(\mu_h+\mu_{h_I}+\gamma_h\right)} & 0 & \frac{1}{\left(\mu_h+\mu_{h_I}+\gamma_h\right)} \end{bmatrix}$$

Thus giving

$$FV^{-1} = \begin{bmatrix} \frac{\beta_{rr}}{(\gamma_r+\mu_r)}\frac{S_r}{N_r} & 0 & 0 & 0 \\ \frac{\beta_{rh}}{(\gamma_r+\mu_r)}\frac{S_h}{N_h} & \left(\frac{p\nu\beta_{hh}}{(\mu_h+\gamma_h)(\nu+\mu_h)} + \frac{(1-p)\nu\beta_{hh}}{(\nu+\mu_h)\left(\mu_{h_I}+\mu_h+\gamma_h\right)}\right)\frac{S_h}{N_h} & \frac{\beta_{hh}}{\mu_h+\gamma_h}\frac{S_h}{N_h} & \frac{\beta_{hh}}{\left(\mu_h+\mu_{h_I}+\gamma_h\right)}\frac{S_h}{N_h} \\ 0 & 0 & 0 & 0 \\ 0 & 0 & 0 & 0 \end{bmatrix}$$

Therefore the characteristic polynomial of FV^{-1} is

$$\lambda^2(\lambda - R^{rr}(t))(\lambda - R^{hh}(t))$$

This results in two non-zero eigenvalues that can be seen to be the effective reproductive rates at time t:

$$
\begin{aligned}
R^{rr}(t) &= \frac{\beta_{rr}}{(\gamma_r + \mu_r)} \frac{S_r}{N_r} \\
R^{hh}(t) &= \left(\frac{p\nu\beta_{hh}}{(\nu + \mu_h)(\mu_h + \gamma_h)} + \frac{(1-p)\,\nu\beta_{hh}}{(\nu + \mu_h)(\mu_{h_I} + \mu_h + \gamma_h)} \right) \frac{S_h}{N_h}
\end{aligned}
$$

The effective reproduction rate for spillover events from rats to humans,$R^{rh}(t)$ is present in FV^{-1} however not in the characteristic equation.

$$
R^{rh}(t) = \frac{\beta_{rh}}{(\gamma_r + \mu_r)} \frac{S_h}{N_h}
$$

We can then infer that the final calculation for the basic reproduction ratio is in the limit as $S_x \to N_x$ and thus cancelling out the compartments remaining in the terms below.

$$
\begin{aligned}
R_0^{rr} &= \frac{\beta_{rr}}{(\gamma_r + \mu_r)} \\
R_0^{hh} &= \left(\frac{p\nu\beta_{hh}}{(\nu + \mu_h)(\mu_h + \gamma_h)} + \frac{(1-p)\,\nu\beta_{hh}}{(\nu + \mu_h)(\mu_{h_I} + \mu_h + \gamma_h)} \right) \\
R_0^{rh} &= \frac{\beta_{rh}}{(\gamma_r + \mu_r)}
\end{aligned}
$$

2 Results

During the model fit of the model outlined in equation set (3) in **Materials and methods** the sequence of tolerances generated were calculated as in Table A. These may be used as is with the approximate Bayesian computation scheme put forward in Beaumont et al (2009) instead of the altered scheme that was used [1].

The 90% credible intervals of the final generation of parameters accepted in the fitting scheme are shown in Table B. For parameter ϕ, the date of minimum recruitment for the vector *Mastomys natalensis*, converted to dates we obtain 4th June, 7th June and 10th June.

Generation, g	Tolerance
1	215.7
2	188.4
3	163.4
4	147.1
5	133.5
6	124.5
7	118.7
8	115.0
9	112.7
10	111.2
11	110.3
12	109.2

Table A: **Tolerances generated from Algorithm 1 during model fit**

	5%	Median	95 %
s	1.54×10^2	3.25×10^2	6.11×10^3
ϕ	0.418	0.427	0.435
β_{rr}	0.672	0.989	1.47
β_{hh}	3.25×10^{-4}	1.85×10^{-3}	3.19×10^{-3}
β_{rh}	33.6	34.8	36.2
$I_r(0)$	0.116	0.156	0.199
$R^{rr}(0)$	45.1	66.3	98.6
$R^{hh}(0)$	3.11×10^{-3}	1.82×10^{-2}	3.09×10^{-2}

Table B: **Credible intervals of 90% and medians for all tested parameters and expected reproduction ratios.**

3 Sensitivity Analysis

We conducted a sensitivity analysis on the proportion of the human population that had previously encountered Lassa fever and the assumed reporting rates for the 2018 epidemic. With the second, we also scaled the initial number of infected, exposed and asymptomatic humans as outlined in the methods. We refit the model using 10% and 20% initially recovered humans after the original assumption of 30% which were within the range of studies that had previously sampled serology in Nigeria and west Africa [2, 3, 4, 5]. We also retested when assuming that the reporting rate for 2018 confirmed case data was 50% and 33% of the true value, while in the original fit we assumed it was 100%.

We investigated for each pair of assumptions of $R_h(0)$ and reporting rate and reran the standard SMC algorithm from Beaumont et al (2009) with the sequence of tolerances produced in Table A [6]. Some of these were unable to complete all 12 generations. When scaling the data and initial values in response to the reporting rates assumed the model we saw that when assuming a 50% reporting rate, the fitting could only complete up to the eighth generation and when assuming a 33% reporting rate only the sixth generation was completed. It is possible that scaling the data, which in turn scales the model, influenced this outcome. Consequently, the increase in error may not necessarily imply a fundamentally worse fit.

In Fig A the differences in quality of fit are minimal when altering the proportion of the human population that had previously encountered Lf. To achieve this, it appears that the transmission parameters relating to humans have accommodated the change. Since the number of susceptibles increases with fewer recovered persons, the human population is more vulnerable to Lf transmission from any source. Human-to-human transmission β_{hh} decreases and so does rat-to-human transmission β_{rh}. Given that the force of infection for humans, λ_h (shown in eq. (2)), is then multiplied by the number of susceptible humans in the system of equations (3) it is logical that as S_h increases that these parameters must decrease to maintain the same output.

When varying the 2018 confirmed case data we saw that while the errors between the simulated data and the reported/altered data the fits were qualitatively similar to that already obtained. The model replicated the trends in the data over the time period, capturing the magnitude of each epidemic. Fig C shows the comparison of fits to 33% reporting rate with varying proportion of recovered. Fig E shows the simulated data and marginal distributions for 50% reporting rate with $R_h(0) = 0.3 \times N_h(0)$.

We found that the more that the epidemic of 2018 represented that of later years the earlier and less severe the rat epidemic was. When increasing the number of cases for 2018, the transmission rate from rats-to-humans did not significantly increase. Instead, the initial proportion of rats infected increased. Furthermore the day of minimum recruitment became earlier and the rat-to-rat transmission rate decreased. Human-to-human transmission rate also increased. This appears to indicate that to accommodate both the larger and smaller epidemics in the data set, the model requires the initial number of infected vectors to direct the magnitude of the 2018 epidemic, whereas the later epidemics are determined by the timing of the pulse of recruitment and the subsequent infection of the new susceptible rodents.

In Fig C are the simulated data 90% intervals outputted during the test for the different choices of initial number of recovered individuals $R_h(0)$ when the data is corrected for an assumed 33% reporting rate. With the exception of the first epidemic, the fit is largely identical to the original fit. In Fig D the differences in the marginal distributions become apparent. The same pattern for different assumptions on $R_h(0)$ are present but the difference for rat-to-rat transmission is also clear. The 90% credible intervals for the rat-to-rat reproductive ratio, R_0^{rr} is now $20.8 - 53.7, 20.8 - 54.5$ and $20.3 - 52.4$ for $R_h(0)$ being $10\%, 20\%$ and 30% of the population respectively. A notable decrease from the original assumptions and fit. Furthermore, the date of minimum recruitment, ϕ, decreased, with 90% credible intervals of 21st May – 5th June for both 10% and 20% and 20th May – 5th June for 30%. This translates to peak recruitment between the end of November to early December in all cases. This correlates well with known milestones for growth of the rat, gestation and weening both being approximately 3-4 weeks, and breeding occurring at the end of the rainy season in October.

In the case that the number of reported cases were 50% of the true incidence we found that minimum vector recruitment was still earlier than in the null hypothesis that reported cases were accurate, but were later than when assumed to be 33%. We obtained a 27th May – 6th June 90% Credible interval with 2nd of June median for the date of minimum vector recruitment. In combination with changing the initial number of recovered humans the recruitment became gradually became later as was seen with the other assumptions for reporting rate with the $R_h(0) = 0.3 \times N_h(0)$. For $R_h(0) = 0.2 \times N_h(0)$ and $R_h(0) = 0.1 \times N_h(0)$ minimum date of recruitment was 28th may, 2nd June, 6th June and 29th May, 3rd June, 7th June respectively as triplets of $5\%, 50\%$ and 95% quantiles.

We show dynamics obtained in the case of 2018 is assumed to have a 50% reporting rate and scaled to correct the under reporting, see Fig E. For sake of brevity we have not shown all choices of $R_h(0)$ in this combination as the dynamics are similar to that already shown. The choices appear to affect the model independently.

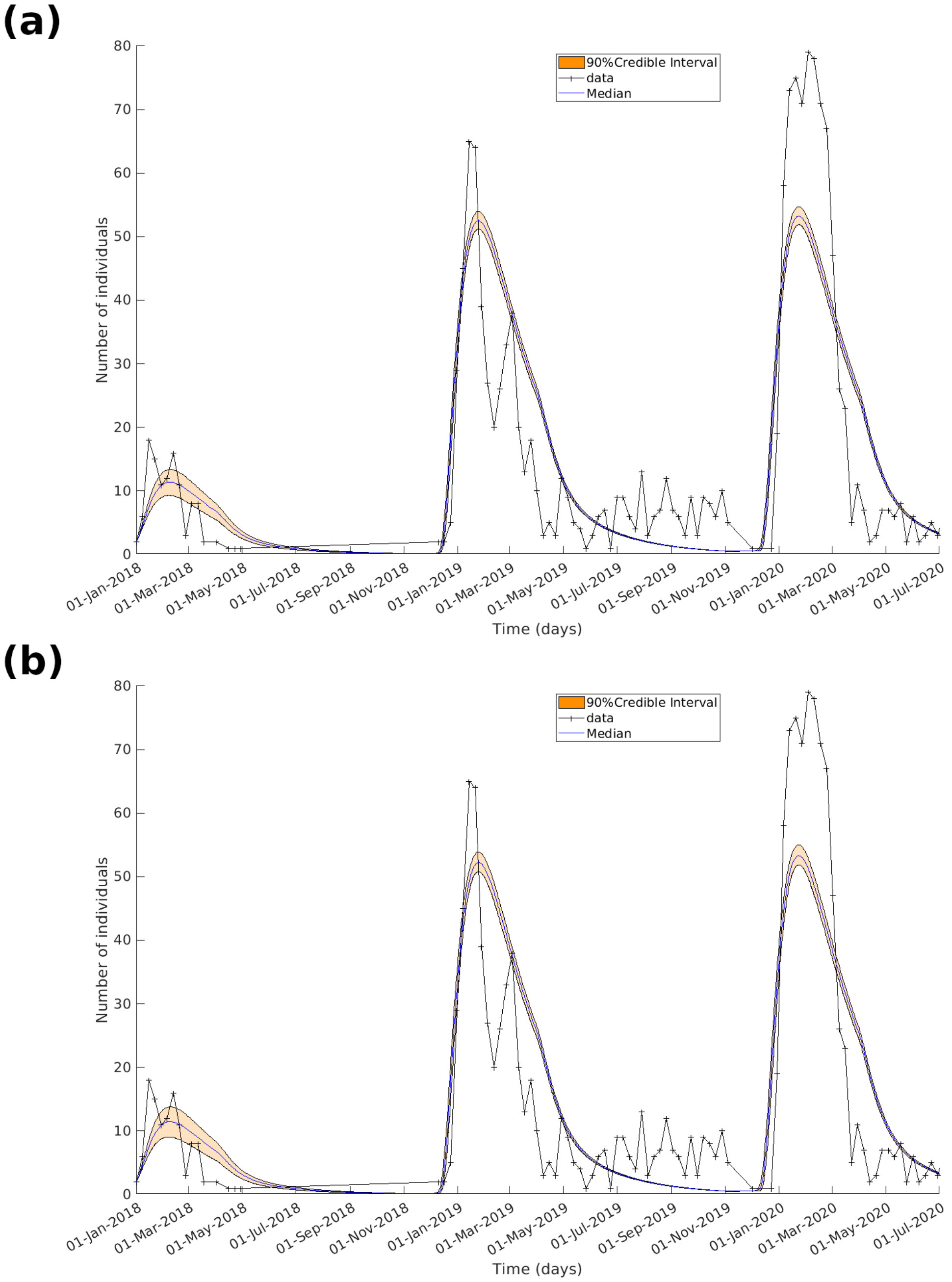

Figure A: **Outputs for simulated number of infected humans compared with observed data.** (a) shows the simulated data output for the model when $R_h(0) = 0.1 \times N_h(0)$. (b) When $R_h(0) = 0.2 \times N_h(0)$. The are qualitatively very similar to that of the original assumption in Fig 3. As previously, in orange is the 90% range of values I_h takes in the final generation at each time point; the median value in blue. Confirmed case data for Nigeria are in black.

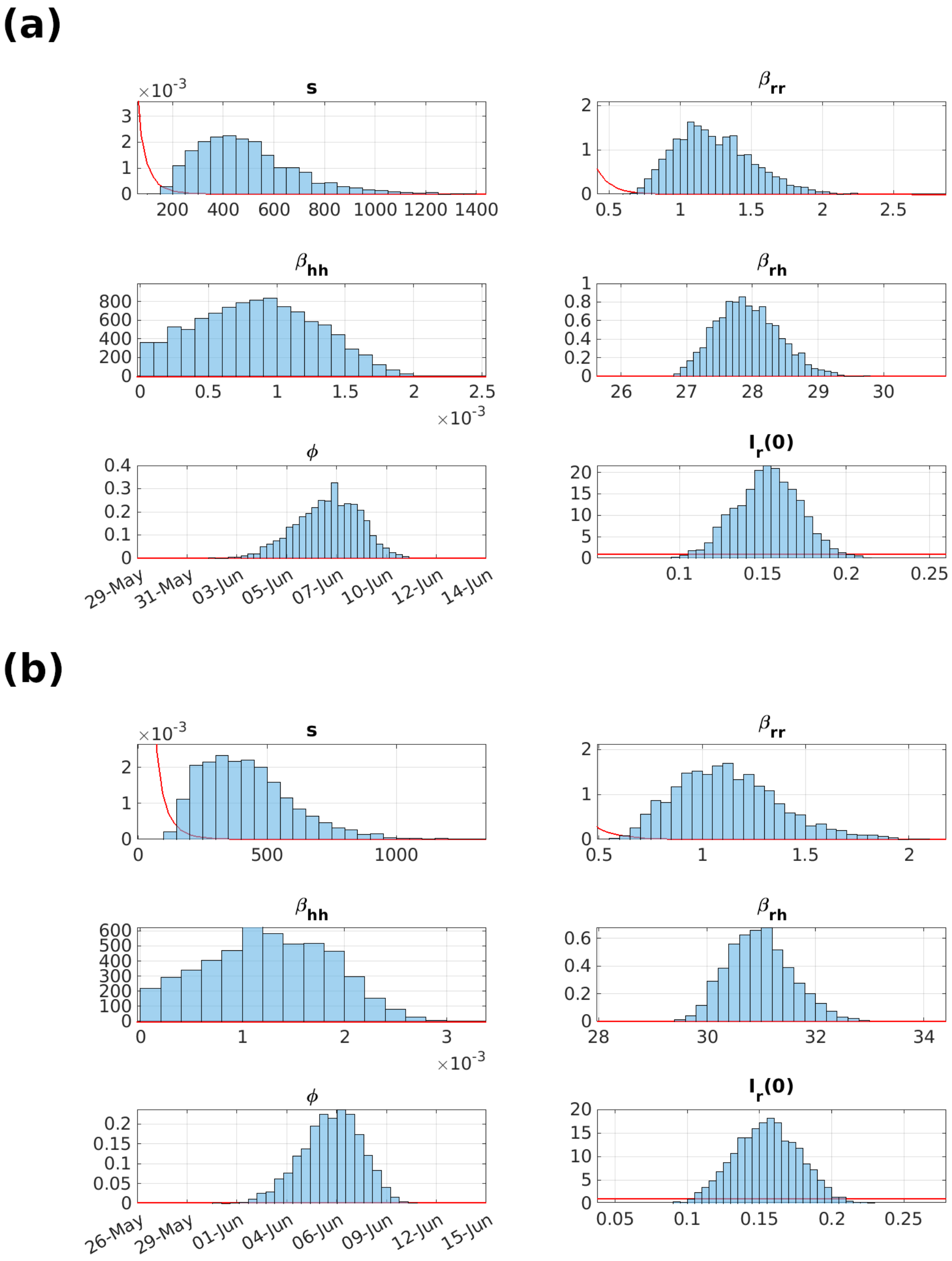

Figure B: **Posterior distributions from sensitivity analysis on $R_h(0)$.** (a) shows the marginal distributions for $R_h(0) = 0.1 \times N_h(0)$. (b) for $R_h(0) = 0.2 \times N_h(0)$. The order is the same as that of Fig 4 with top left the shape parameter of the rodent recruitment function, s. Top right the rodent-to-rodent transmission rate β_{rr}. Mid left the human-to-human transmission rate β_{hh}. Mid right the rodent-to-human transmission rate β_{rh}. Bottom left the date of minimum rat recruitment ϕ. Bottom right the initial proportion of infected rodents, $I_r(0)$.

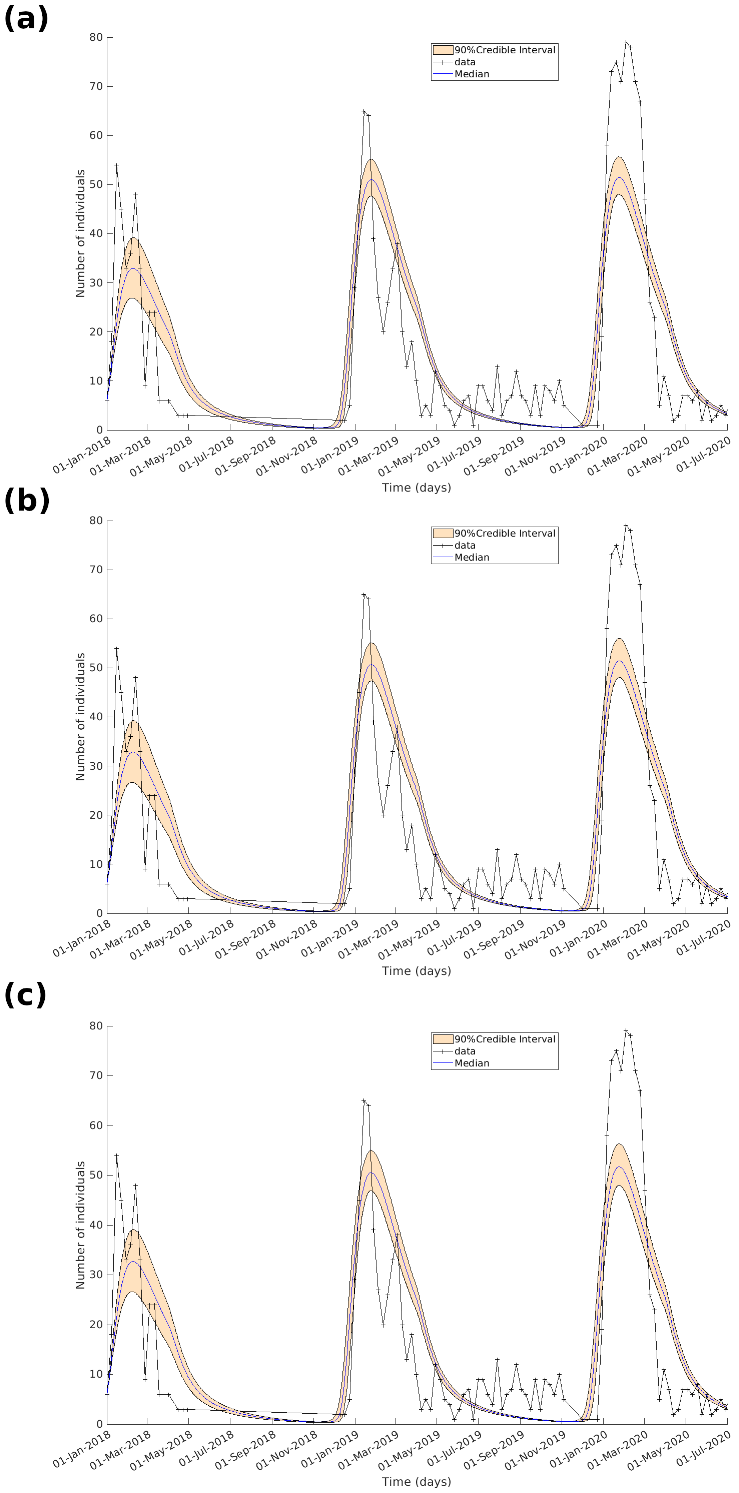

Figure C: 90% **credible interval for simulated data compared with reported cases with 2018 corrected for assumed under reporting at** 33%**.** With data scaled to account for assumed under reporting for 2018 (a) $R_h(0) = 0.1 \times N_h(0)$, (b) $R_h(0) = 0.2 \times N_h(0)$ and (c) $R_h(0) = 0.3 \times N_h(0)$.

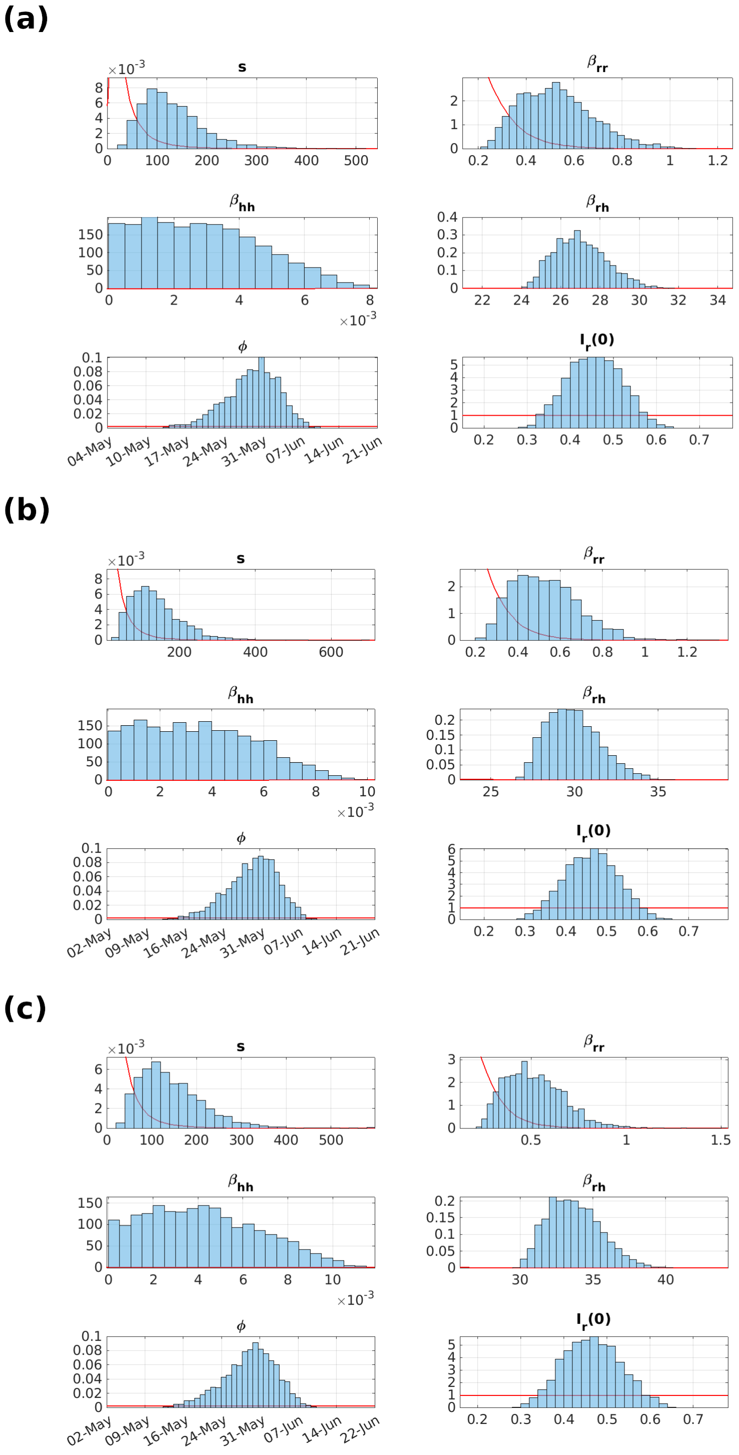

Figure D: **Marginal distributions for fittings with reported cases with 2018 corrected for assumed under reporting at 33%.** With data scaled to account for assumed under reporting for 2018 (a) $R_h(0) = 0.1 \times N_h(0)$, (b) $R_h(0) = 0.2 \times N_h(0)$ and (c) $R_h(0) = 0.3 \times N_h(0)$ all marginal distributions shown in order as previously used.

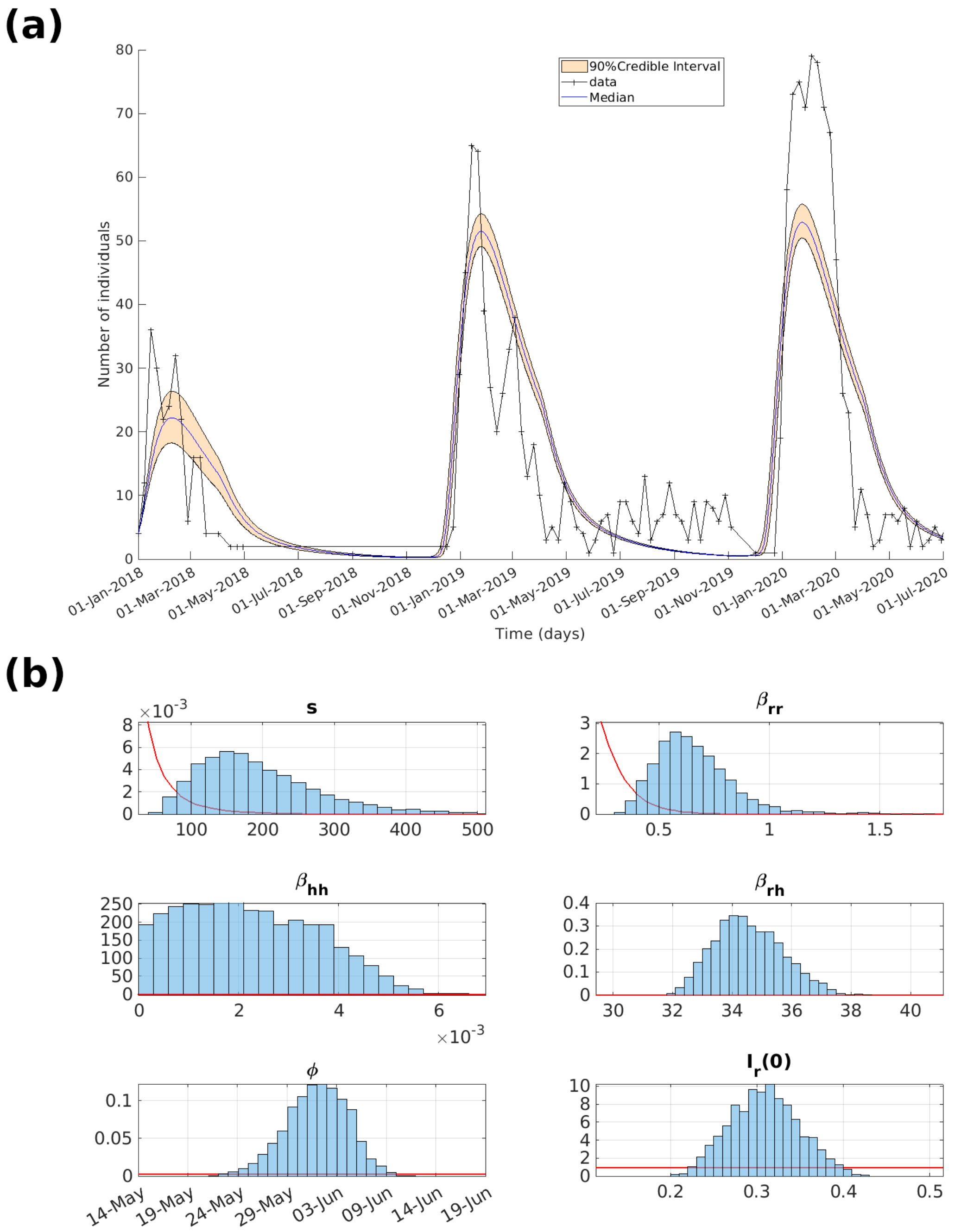

Figure E: **Results for $R_h(0) = 0.3 \times N_h(0)$ and** 50% **reporting rate (a)** 90% **Credible interval for simulated data (b) Marginal posterior distributions.**

References

[1] Peel AJ, Pulliam J, Luis A, Plowright R, O'Shea T, Hayman D, et al. The effect of seasonal birth pulses on pathogen persistence in wild mammal populations. Proceedings of the Royal Society B: Biological Sciences. 2014;281(1786):20132962.

[2] Keenlyside RA, McCormick JB, Webb PA, Smith E, Elliott L, Johnson KM. Case-control study of Mastomys natalensis and humans in Lassa virus-infected households in Sierra Leone. The American journal of tropical medicine and hygiene. 1983;32(4):829–837.

[3] Ter Meulen J, Lukashevich I, Sidibe K, Inapogui A, Marx M, Dorlemann A, et al. Hunting of peridomestic rodents and consumption of their meat as possible risk factors for rodent-to-human transmission of Lassa virus in the Republic of Guinea. American Journal of Tropical Medicine and Hygiene. 1996;55:661–666.

[4] Tomori O, Fabiyi A, Sorungbe A, Smith A, McCormick JB. Viral hemorrhagic fever antibodies in Nigerian populations. The American journal of tropical medicine and hygiene. 1988;38(2):407–410.

[5] Tobin EA, Asogun D, Akpede N, Adomeh D, Odia I, Gunther S, et al. Lassa fever in Nigeria: Insights into seroprevalence and risk factors in rural Edo State: A pilot study. Journal of Medicine in the Tropics. 2015;17(2):51.

[6] Beaumont MA, Cornuet JM, Marin JM, Robert CP. Adaptive approximate Bayesian computation. Biometrika. 2009;96(4):983–990.
