# Supplementary material for: Modelling seasonality of Lassa fever incidences and vector dynamics in Nigeria
Source: PLoS Negl Trop Dis. 2023 Nov 13;17(11):e0011543. doi: 10.1371/journal.pntd.0011543 (PMC10681316; doi:10.1371/journal.pntd.0011543)
Supplement: Attachment — Submitted filename: PNTD-D-23-00927.docx [file pntd.0011543.s002.docx]

Summary

The authors present a (non-age-stratified) compartmental ordinary differential equation model for the dynamics of Lassa fever in Nigeria. The key features of the model are that it is vector-based ODE model that is primarily driven by the vector. The model successfully reproduces seasonal epidemics observed from 2018 onwards. Overall, the paper is well written and engaging and I think that the model has potential, however, there is one critical issue that needs to be addressed before publication.

1. The initial number of recovered individuals is assumed to be 0 (see page 6, lines 163-4). Given that seroprevalence data are cited from 2015-era publications, it is not realistic that (nearly) the entire population would be naïve in 2018. I would expect that the model would be very sensitive to the initial number of recovered individuals. Unfortunately, no sensitivity analysis around this parameter was investigated. This is even though sensitivity analyses were performed for much less critical parameters, see comments below. Without knowing how the number of initial recovered individuals influences the model outputs, I don’t think that any useful conclusions can be drawn. It is common for epidemics in naïve populations to display “waves” of infection. Thus, how do we know that the model is reproducing the seasonal epidemics because a naïve population is being modelled, or because the model is capturing the essential nature of the disease dynamics? I would suggest either including a sensitivity analysis for the number of initial recovered individuals, or alternatively adding it to the calibration parameter set. One way or another, this manuscript should not be published without resolving this issue in a satisfactory manner.
2. I am not clear on why the authors expect the model to be sensitive to the size of the rat population. From the model equations, it seems likely that setting the rat population to an arbitrary level and then scaling the transmission coefficient \beta_{rr} (which would happen automatically during the calibration procedure) should be sufficient for the purposes of this model. While it is useful to perform the sensitivity analysis anyways (in case of any “surprises”), I would not spend any time on this issue in the main manuscript (since no such “surprises” arise). Instead, I would strongly suggest addressing the issue discussed in point (1), which is of much more import to the overall model dynamics and the ability to draw conclusions from the results. Also, on page 6, lines 167-168 it looks like there is a typo “1, 10^6” should be “1x10^6”? N(0)=1 doesn’t seem to appear further on in the text. As another related, but minor, issue, why not take the initial values for the rat population from the stable periodic solution of the rat-specific equations (evaluated at the appropriate time)? This would simplify the initial parameter set and seems much more realistic.
3. The case fatality rate (Page 5, lines 151-156:) is equal to the flow out of the Infected class due to disease-related deaths divided by the total flow out of the compartment, i.e., CFR = \mu_{h_I} / (\gamma_h + \mu_{h_I} + \mu_h). With the parameters and CFR as reported in the manuscript this would yield \mu_{h_I} = 0.0242 per day vs 0.0195 per day (about a 25% difference). This would probably not make a huge difference to the disease dynamics, but it will make a large difference when investigating the change in deaths due to disease after an intervention has been introduced. I would correct this issue here, rather than it become an issue in subsequent models.

Minot comments

- Page 3, lines 83-84: “All humans are assumed to have an average life expectancy of 1/\mu_h days regardless of their compartment.” This statement is not entirely true, since Infected individuals experience additional mortality beyond the background mortality rate. Suggestions: delete “regardless of their compartment”.
- Page 3, lines 84-85: “That is the number of individuals in each compartment decrease at a rate of \mu_h per capita.” – This statement is very confusing and not entirely true. It is only true in the case where there are no births and no infection dynamics, i.e., in the absence of any model for Lf. I would suggest just deleting this sentence.
