# Supplementary material for: Modelling seasonality of Lassa fever incidences and vector dynamics in Nigeria
Source: PLoS Negl Trop Dis. 2023 Nov 13;17(11):e0011543. doi: 10.1371/journal.pntd.0011543 (PMC10681316; doi:10.1371/journal.pntd.0011543)
Supplement: Attachment — Submitted filename: Review_Reply.odt [file pntd.0011543.s003.odt]

Editors: Dr Chukwunonso Nzelu and Andrea Marzi
Dear editors and reviewers,
We would like to extend our sincere gratitude to the editors and the reviewers for their valuable time and constructive feedback on our paper titled "Modelling seasonality of Lassa fever in Nigeria". Your insights and comments have been instrumental in enhancing the quality of this work, and we genuinely appreciate your efforts in helping us refine it for publication.
General response to all reviewers
In response to your comments, we have adjusted the model dynamics, refitted the model and conducted additional sensitivity analyses on important parameters and assumed initial values. We have re-examined the conclusion and clarified certain points accordingly. 
In particular, we have clarified that our vector model sought to represent seasonal fluctuations in rat populations in close proximity to humans, and does not represent a prediction for the entire rat population of Nigeria. Additionally, our sensitivity analyses now focus on the initial proportion of humans that have previously been exposed, and are now immune, to Lassa fever. The summary of the sensitivity analysis in the discussion section has been revised accordingly. 
Below, we have responded to each reviewer’s individual comments.
Responses to reviewers’ individual remarks:
Reviewer 1: 
The authors built and tested an ABC model to explore Lassa seasonality in Nigeria. The paper is very well written and easy to understand. I have specific questions below, most on the vector modeling, but my overview is that the authors argue that vector reproduction seasonality drives Lf in humans yet they admit their vector model is quite generalized. I agree that the vector model used here is somewhat primitive and the observed dynamics are unrealistic. This leads to unrealistic fitted parameters, including R0 values the authors state were 'well above that which one would consider to be an extremely infective disease' but left out of the main text (seen in table S2). A vector R0 of 135-1030 seems high, and aligns with my comments below on LASV rat transmission being too high and the population dynamics unsustainable. My suggestions for the authors are to take their own comments (lines 351-371) and improve their vector transmission model to hone in on more appropriate parameters.
We would like to thank the reviewer for their comments and suggestions. We have altered the model’s parameters and some prior distributions in response to the critique. In addition to clarifying the definition of the vector within our vector model as mentioned above in our general response, we have now included seasonal variation in vector transmission rates as a result of observed vector behavioural changes linked with seasonal migration of the vectors during the dry season. Specifically, the rat-to-human transmission parameter, beta_rh, is now doubled between November to March to account for the increased contact between vectors and humans that has been displayed in papers such as [1]. The refitted results produce a 90% Credible Interval for vector R0 of 45–98. While still large, it is not unusual for vector R0 estimates to be large when considering spatio-temporal heterogeneity in vector dynamics [2, 3]. We believe that the work presented here follows sound methodology and meaningful conclusions were reached from the work. We have identified several avenues for future work in the discussion of our paper that will further refine our modelling work. [
I was also a bit confused as [the] authors referenced fig5a and 5b, but there were only 4 figures in the manuscript…
We apologise for this error. The figures have been redrawn following the above inclusion of seasonal rat-to-human contact rates, and they have now been labelled correctly. 
Line 9: LASF? Is this supposed to be Lf or LASV?
This is correct. We have now corrected this typo. [Line 9]
line 16: years since 2017, yet abstract says 2018? Was it introduced in 2017 and 2018 was the first 'bad' year?
Our apologies for the confusion. We have clarified in the text [Line ] that while surveillance efforts started prior to 2018 and also saw similar patterns in case incidence, we have excluded them. Ilori et al (2019) outlines Nigerian surveillance efforts and the significant increase in diagnostic capacity available in 2018 that was not available in previous years [4]. It was observed that cases increased in previous years similar to that seen in the data presented; however, the data we have been provided with starts at 2018 and the 2018 epidemic was significantly larger than those of 2016 and 2017. We would likely need to account for the difference in diagnostic capacity if previous years were included. [Lines 16-17, 80-85]
line 19: M. natalensis, rather than Mastomys?
Thank you. We have now corrected this. [Line 22]
line 20: Maybe state Mastomys natalensis is a rat prior to just coming across it here?
Line 4 has been changed to introduce M. natalensis as a rat.
lines 24-26: rat bites? rat feces? fomite transmission?
The transmission methods have now been included explicitly, namely food contamination through faeces and urine, and consumption of the rats themselves. [Lines 29-30]
line 68: population dynamics of Mastomys natalensis?
Correct, we have altered the line. [Line 96]
line 82: Do asymptomatic individuals recover at the same rate?
This is correct and we have now clarified this in the text. [Line 111]
line 86: Are recovered individuals (R) assumed to have protective immunity? Same for the rats?
This is correct. We have now clarified this within our description of the model. [Lines 112, 127]
lines 91-92: all infected rats are thus asymptomatic?
Correct. This has now been clarified within the manuscript. [Lines 124-125]
line 100: How do you keep the reservoir population constant with seasonal breeding and constant deaths? Do you mean the avg population from year to year?
We have clarified in the manuscript that the population was stationary from year to year and was not assumed to be constant. [Line 134]
line 108: also betarr? How was betarr parameterized? What is the seasonal distribution of Ir (peak vs off season)?
We’ve added a description of how beta_rr was parameterised. There are not many studies capturing the seasonality in the prevalence of viremia in rats, modelled by Ir, however Fichet et al. (2007) found that it is high in the wet season and lower in the dry season [1]. This is in contrast to that observed in our model but serves to highlight what is not understood about the infection cycle of the Lf. In our model, the viremic proportion of the vector varies greatly, increasing to nearly 70% at its peak. These large proportions are comparable to localised samples in other studies such as Kenlyside et al. (1983) and Safronetz et al. (2013) [5, 6]. Furthermore, it is noted in Fichet et al. (2014) that rats in locations that are considered to be high-endemic have higher prevalences [7]. Since most cases are spillover events in high-endemic areas of Nigeria one concludes that the model which assumes homogeneous mixing on a national scale is replicating dynamics closer to that of high endemic areas. [Lines 140-142, 428-430]
line 113: Is there an assumption that no human to rat transmission occurs?
This is correct. We have clarified this within the methodology. [Lines 142-145]
line 154: Others (Bausch D.G., et al. Lassa fever in Guinea: I. Epidemiology of human disease and clinical observations. Vector Borne Zoonotic Dis. 2001;1(4):269–281)) have found CFRs to be as high as 50%, why is the assumed CFT so low here? Is it possible a large number of unreported deaths occurred?
Thank you for this insight. It is possible that deaths are under-reported however it is broadly accepted that the CFR for Lassa in broader demographics is approximately 20% [8, 9]. Here we used the CFR as reported in the data, so we have kept this value to maintain consistency. We have clarified this within the manuscript. [Lines 199-201]
lines 157-159: You are also assuming 100% reporting. I imagine early reporting was likely bad, even if individuals were symptomatic. How do the parameters change if you estimate a higher number of infected individuals initially? I'm interested in the model sensitivity.
It has been noted that the symptoms of Lassa fever are difficult to differentiate from other febrile illnesses and so it is likely that the case numbers are often under reported. We have included in the sensitivity analysis an investigation on how the model dynamics and fitting changes without the assumption of 100% reporting for 2018. We investigate the model under assumptions of 50% and 33% reporting, altering the initial number of infected individuals for 2018, and thus demonstrating the effect of adjusting the initial number of infected individuals on parameter estimates. [Lines 361-383]
line 168: Which population size of rats is used for the model results/figures?
We have changed the focus of the sensitivity analysis and now only use one population for rats. Thus there is no longer need for clarification.
line 283: The figure numbering seems off. Fig 4 is vector dynamics, fig 4b is the timeline of vector epidemics, but there are references and a caption for a figure 5?
We apologise for this error. We have now relabelled the figures and their captions, and fixed the references to figures.
How does human to human transmission occur?
Human-to-human transmission occurs by transferral of bodily fluids, normally within hospital settings without adequate ppe [8]. We have noted this within the manuscript in the introduction section. [Lines 30-33]
Fig 4: It seems the infected vector has the same seasonality of LASV as humans. Isn't it more likely LASV transmission increases in rats during their breeding season (Nov-Jan?), and then there is some exposed period in rats prior to fecal shedding and the increase in cases in humans? Is there no lag?
We have revisited our description of the model, taking care to note that we are modelling the number of rats in close proximity to humans. Therefore the model reflects seasonal recruitment into rural homes rather than births. We have also revisited the transmission rate for rat-to-human interactions however can find no literature to suggest an alternative seasonal function from the constant rat-to-rat transmission that is common in compartmental modelling. It is possible that this is the case however we have not incorporated an investigation of this into our model since this is outside the scope of our analysis, however, it would provide an interesting avenue for future work. [Lines 118-119, 151-155]
We have assumed that there is no lag between transmission and infectiousness for the rats as it appears small or insignificant and an exposed compartment for rats has not been assumed in previous studies [10-17]. We have clarified this within the methodology. Again, this could be true, however, we believe further investigation into the pathology of LASV and transmission within the zoonotic vector would be necessary to provide evidence to alter the mechanistic approach for the model. [Lines 125-127] 
Fig4: Why does it look like there are 0 susceptible rats for most of the year?, it's not clear to me why they peak directly before infections occur. Are you assuming 100% of rats are exposed to LASV every year and the only reason Lf drops in humans is because of vector susceptible burnout?
The resulting fit of the model parameters indicate that the vast majority of the rodents that are recruited into close proximity with humans become infected and then cause the increase in spillover to humans. Assuming 100% of rats are exposed to LASV every year was not an explicit assumption in our model, however, and is only the simulated outcome of the model under the constraints of fitting and model design. We have adjusted figure 4 (now figure 5) to instead emphasise the relative proportions of susceptible and infected (local) rats throughout the study period. [Figure 5]
Fig4 & lines 351-371: How does the Ir population exceed the annual Sr population if Rr never become susceptible? Does transmission in rats happen so quickly that all newborn rats are immediately infected? Then only the very low number of infected individuals the following year seeds the entire new generation of rats? It is more likely the seasonal reproduction isn't so strong and there is a higher number of infected rats throughout the year. Even if the reproduction seasonality is this strong, it seems highly unlikely 100% of newborns are infected and the vector circulation nearly dies out every year. Whether that means a longer infected period, some sort of latent (E) period, or lowered infectiousness I don't know. The dynamics seen in Fig 4 seem unrealistic. Seasonal reproduction has variation, and you are one 'bad' or 'late' reproductive season away from eliminating LASV in the vector. I see you have addressed some of the issues with the vector population in the lines listed in the results. I think the authors need to expand and address some of the above issues. The authors need to expand because some of the fitted parameters are likely going to be inaccurate if a more accurate vector population model is used.
The model uses a recruitment function and so the model shows that juveniles that have been weaned and moved into relevant areas, i.e., close or inside rural homes where contact with people is more regular, have become infected. This recruitment would also attempt to model migration and changes in behaviour seen in the seasonal habits of M. natalensis. Therefore, it may not be the small number of susceptible survivors that seed the next year. This has been now clarified in the manuscript as stated in the general response to reviewers above. [Table 2, Lines 53,127, 404-407]
We have not found in the literature conclusive modelling of the total population of the vector. We have found conformity on seasonal reproduction (end of the rainy season) with varying degrees of seasonality (varying between nearly all breeding focused in the rainy season, to less severe, year-out breeding but still favouring the end of the rainy season) based on region of study [18-25]. When the new-borne rats become involved in the infection dynamics is less clear since it is approximately one month from birth when they are weaned but migration from likely nesting habitat to rural homes is not as well documented. While hypothesised that the rodents migrate towards rural homes during the harvest and dry season it is not so conclusive in tracking studies [26, 27]. [Lines 417-426]
As a result it is difficult to quantify and disentangle the relevant factors on the fundamental problem with Lassa fever in Nigeria of how spillover changes between seasons. We are currently unable to develop a complete understanding but hope to illustrate the importance of disentangling the vector-human contact rate, true prevalence values and rodent population cycles to identify risk factors of Lf in Nigeria. While we make advances in the mechanics modelled, we have also fitted to the 3 consecutive epidemics rather than a single outbreak in an attempt to elucidate transmission dynamics that might be missed in their separation. This constraint and methodology results in the dynamics shown and should initiate more investigation into the transmission dynamics of Lassa fever. [Lines 388-397]
line 365: I'm not sure a metapopulation model would be appropriate because you have country-level reporting. If you had higher resolution data this may be possible.
Case data at a higher-resolution than presented is now available, and we are currently developing and analysing results from a metapopulation framework to answer different questions about Lf transmission than presented here. We feel the national-scale work as presented here is novel and of sufficient interest in and of itself. [Lines 459-462]
conclusion: The authors conclude that seasonal vector reproduction dynamics drive Lf in humans. This comes directly after 20 lines (351-371) in the results explaining that their vector model is likely inaccurate and needs to be improved. It is already known that M. natalensis is the primary driver of Lf.
We thank the reviewer for their thorough and useful review. We acknowledge the limitations of our work however we believe we have valid contributions to the literature. We have extended previous models and have fitted to an extended period of time covering multiple epidemics. We intend to show that the pattern in seasonality can be explained with reasonably assumed population recruitment dynamics and what the model would require of the population to simulate the data observed. It provides a link between the population cycle of the vector and that of the incidence profile of Nigeria over the period.
line 375: Do the rats get Lf or LASV?
The rats only get LASV and do not suffer the infection. This has been changed in the manuscript. [Line 471]
Reviewer 2:
The authors present a (non-age-stratified) compartmental ordinary differential equation model for the dynamics of Lassa fever in Nigeria. The key features of the model are that it is a vector-based ODE model that is primarily driven by the vector. The model successfully reproduces seasonal epidemics observed from 2018 onwards. Overall, the paper is well written and engaging and I think that the model has potential, however, there is one critical issue that needs to be addressed before publication.
1. The initial number of recovered individuals is assumed to be 0 (see page 6, lines 163-4). Given that seroprevalence data are cited from 2015-era publications, it is not realistic that (nearly) the entire population would be naïve in 2018. I would expect that the model would be very sensitive to the initial number of recovered individuals. Unfortunately, no sensitivity analysis around this parameter was investigated. This is even though sensitivity analyses were performed for much less critical parameters, see comments below. Without knowing how the number of initial recovered individuals influences the model outputs, I don’t think that any useful conclusions can be drawn. It is common for epidemics in naïve populations to display “waves” of infection. Thus, how do we know that the model is reproducing the seasonal epidemics because a naïve population is being modelled, or because the model is capturing the essential nature of the disease dynamics? I would suggest either including a sensitivity analysis for the number of initial recovered individuals, or alternatively adding it to the calibration parameter set. One way or another, this manuscript should not be published without resolving this issue in a satisfactory manner.
We thank the reviewer for their time and review. We have changed our sensitivity analysis and now include the initial proportion of the population that is recovered and immune to Lassa fever. Here, we now assume 30% of Nigeria has been in contact with LASV and thus are protected from it as a baseline scenario, and investigate the effect of assuming 20% and 10% immunity at the start of the study period as seen in estimates from previous works [5,28-30]. Although some samples have seen higher, there are many factors involved and high-endemic areas exist for Lf which may not be a complete representation of Nigeria as a whole, as we have modelled here [30]. We see that the model is still able to replicate the annual trends with the same quality of fit (i.e. the same tolerance for error between data and simulated data) and a decrease of rate of zoonotic transmission as more of the population was susceptible to infection. [Page 10, Table 3 and Line 349-360]
2. I am not clear on why the authors expect the model to be sensitive to the size of the rat population. From the model equations, it seems likely that setting the rat population to an arbitrary level and then scaling the transmission coefficient \beta_{rr} (which would happen automatically during the calibration procedure) should be sufficient for the purposes of this model. While it is useful to perform the sensitivity analysis anyways (in case of any “surprises”), I would not spend any time on this issue in the main manuscript (since no such “surprises” arise). Instead, I would strongly suggest addressing the issue discussed in point (1), which is of much more [importance] to the overall model dynamics and the ability to draw conclusions from the results. Also, on page 6, lines 167-168 it looks like there is a typo “1, 10^6” should be “1x10^6”? N(0)=1 doesn’t seem to appear further on in the text. As another related, but minor, issue, why not take the initial values for the rat population from the stable periodic solution of the rat-specific equations (evaluated at the appropriate time)? This would simplify the initial parameter set and seems much more realistic.
Thank you for your detailed comment. We agree that the initial proportion of recovered individuals is of much more importance to the overall model dynamics and parameter estimates. We have refitted the model with the necessary alterations as stated above in addressing the reviewer’s first point, allowing us to focus sensitivity analyses toward the  initial proportion of recovered and immune persons.
As for the initial number of M. natalensis in the system, N_r(0), it should have been 1 and 1x10^6 however the sensitivity analysis is no longer focused on this aspect of the model and has been removed. With respect to the initial values for the rat population, the endemic equilibrium is not always positive due to the time-dependent recruitment function with some choices of parameter values. Therefore we’ve included the initial value as part of the fitting process. [Lines 221-223]
3. The case fatality rate (Page 5, lines 151-156:) is equal to the flow out of the Infected class due to disease-related deaths divided by the total flow out of the compartment, i.e., CFR = \mu_{h_I} / (\gamma_h + \mu_{h_I} + \mu_h). With the parameters and CFR as reported in the manuscript this would yield \mu_{h_I} = 0.0242 per day vs 0.0195 per day (about a 25% difference). This would probably not make a huge difference to the disease dynamics, but it will make a large difference when investigating the change in deaths due to disease after an intervention has been introduced. I would correct this issue here, rather than it become an issue in subsequent model
We are grateful for the reviewer’s correction and have implemented their change into the model. [Lines 196-201]
- Page 3, lines 83-84: “All humans are assumed to have an average life expectancy of 1/\mu_h days regardless of their compartment.” This statement is not entirely true, since Infected individuals experience additional mortality beyond the background mortality rate. Suggestions: delete “regardless of their compartment”.
Thank you for clarifying this. We have deleted the relevant statement.
- Page 3, lines 84-85: “That is the number of individuals in each compartment decrease at a rate of \mu_h per capita.” – This statement is very confusing and not entirely true. It is only true in the case where there are no births and no infection dynamics, i.e., in the absence of any model for Lf. I would suggest just deleting this sentence.
The sentence has been changed and an alternative description of the parameter has been included. [Lines 113-114]
The model does reproduce the observed epidemic pattern, but it is not clear how much of this is due to the assumption of a naive initial population. I don't think that the claim can be made (yet) that the model reproduces the observe epidemic behaviour because is captures the essential features of the dynamic process. In other words, I don't think that any useful conclusions can be drawn until the issues in the Methods section are resolved.
We thank the reviewer for their comments which have shaped our work. We have investigated the effect of the initial recovered population in our sensitivity analysis. We believe this addresses the reviewer’s concerns.
Reviewer 3:
The objectives of the study are clearly described and the model proposed is described in detail. The literature review is well-developed. There is a clear motivation for the statistical estimation procedure adopted.
We thank the reviewer for their time and comments, we have taken each seriously and added to our manuscript.
Does the analysis presented match the analysis plan? Yes
Are the results clearly and completely presented? Could be improved in the discussion and interpretation, but I think they are fine.
In response to reviewers’ comments we have revisited the discussion, adding more detail that has been outlined in this response to reviewers’ comments.
Are the figures (Tables, Images) of sufficient quality for clarity? Figures could be surely improved. They do not seem of very good quality.
We apologise for the lack of clarity on the figures. We are unsure as to why the figure quality was poor. We have increased the resolution of the figures in hopes of alleviating this issue.
Are the conclusions supported by the data presented? Yes.
Are the limitations of analysis clearly described? Could be improved
The paper is interesting and well-written. The methodology adopted seems adequate and developed with care.
We thank the reviewer for their comments and time. We have revisited our limitations section within our discussion in response to comments made during review.
The only concern that I have about the model proposed is that it does not seem to capture the increasing level of seasonality through the years. The seasonal behavior in 2018 is quite low, starting to increase evidently in 2019 and even more in 2020. Apparently, there is an evolution in the seasonal behavior, which the model seems not able to capture. Is this a neglectable aspect of the process or should we care about it? Should we expect that in the years 2021, 2022, and 2023 this seasonal behavior will be even stronger?
We have previously remarked in this response that 2018 was the first year of operation of new laboratories capable of diagnosing LASV [4]. The number of cases likely increased in the following years as familiarity with the processes involved and as such the level of surveillance was improved. [Line 75-86]
We have included in the sensitivity analysis an alternate view of the data where we assume that only a proportion of the data from 2018 was collected. We fitted the model to the altered data to investigate the model's capability of accommodating the changes year-on-year. This should abate doubts on whether model dynamics and parameters in the primary fitting are heavily dependent on the initial conditions of a year which we suspect to be under-reported. [Line 371-383]
By the way, why did the authors use just the data from 2018 to 2020?
In this study we wished to extend understanding on modelling Lassa fever by modelling over multiple epidemics to investigate underlying seasonal dynamics of the vector and spillover events. We had access to data from 2018 onwards, and for the 2021 season we suspect that nationwide interventions during the COVID pandemic affected reporting as we see a more than a 50% decrease in cases from 2020 [31]. We thus chose not to include this season during our study. [Line 75-86]
I understand that there could be problems with the availability of more recent information, but this should be better explained in the data description.
We have included justification for not including more recent information in the introduction and methodology sections of the manuscript. [Line 73-86]

Also, I would suggest the authors display the data at some point at the beginning of the paper, because when I was reading I was expecting to see a graphical display of the problem. Seeing it just in the results section is probably too late.
Thank you for your insight. We have now presented data in the methodology section as figure 1. [Fig 1]

Thank you for considering our manuscript with PLOS Neglected Tropical Diseases.
Yours Sincerely,
James McKendrick, Dr Warren Tennant, Prof. Michael J. Tildesley

[1] Fichet-Calvet E, Lecompte E, Koivogui L, Soropogui B, Dor ́e A, Kourouma F, et al. Fluctuation of abundance and Lassa virus prevalence in Mastomys natalensis in Guinea, West Africa. Vector-Borne and Zoonotic Diseases. 2007;7(2):119–128.
[2] Hartemink N, Purse B, Meiswinkel R, Brown H, De Koeijer A, Elbers A, et al. Mapping the basic reproduction number (R0) for vector-borne diseases: a case study on bluetongue virus. Epidemics. 2009;1(3):153–161.
[3] Smith DL, McKenzie FE, Snow RW, Hay SI. Revisiting the basic reproductive number for malaria and its implications for malaria control. PLoS biology. 2007;5(3):e42.
[4] Ilori EA, Frank C, Dan-Nwafor CC, Ipadeola O, Krings A, Ukponu W, et al. Increase in lassa fever cases in Nigeria, January–March 2018. Emerging infectious diseases. 2019;25(5):1026.
[5] Keenlyside RA, McCormick JB, Webb PA, Smith E, Elliott L, Johnson KM. Case-control study of Mastomys natalensis and humans in Lassa virus-infected households in Sierra Leone. The American journal of tropical medicine and hygiene. 1983;32(4):829–837.
[6] Safronetz D, Sogoba N, Lopez JE, Maiga O, Dahlstrom E, Zivcec M, et al. Geographic distribution and genetic characterization of Lassa virus in sub-Saharan Mali. PLoS neglected tropical diseases. 2013;7(12):e2582.
[7] Fichet-Calvet E, Becker-Ziaja B, Koivogui L, G ̈unther S. Lassa serology in natural populations of rodents and horizontal transmission. Vector-Borne and Zoonotic Diseases. 2014;14(9):665–674.
[8] World Health Organization. Lassa fever fact sheet, 31 July 2017; © 2023 WHO. Available from https://www.who.int/news-room/fact-sheets/detail/lassa-fever.
[9] Nigeria Centre for Disease~Control and Prevention. An update of Lassa fever outbreak in Nigeria: situation reports. Available from https://ncdc.gov.ng/diseases/sitreps/?cat=5&name=An%20update%20of%20Lassa%20fever%20outbreak%20in%20Nigeria
[10] Musa SS, Zhao S, Gao D, Lin Q, Chowell G, He D. Mechanistic modelling of the large-scale Lassa fever epidemics in Nigeria from 2016 to 2019. Journal of theoretical biology. 2020;493:110209.
[11] Zhao S, Musa SS, Fu H, He D, Qin J. Large-scale Lassa fever outbreaks in Nigeria: quantifying the association between disease reproduction number and local rainfall. Epidemiology & Infection. 2020;148:e4.
[12] Collins OC, Duffy KJ. Using Data of a Lassa Fever Epidemic in Nigeria: A Mathematical Model Is Shown to Capture the Dynamics and Point to Possible Control Methods. Mathematics. 2023;11(5):1181.
[13] Ojo MM, Benson TO, Shittu AR, Doungmo Goufo EF. Optimal control and cost-effectiveness analysis for the dynamic modeling of Lassa fever. J Math Comput Sci. 2022;12:Article–ID.
[14] Bakare EA, Are EB, Abolarin O, Osanyinlusi SA, Ngwu B, Ubaka ON. Mathematical modelling and analysis of transmission dynamics of Lassa fever. Journal of Applied Mathematics. 2020;2020:1–18.
[15] Abdullahi MB, Doko UC, Mamuda M. Sensitivity analysis in a Lassa fever deterministic mathematical model. In: AIP conference proceedings. Vol. 1660. AIP Publishing; 2015.
[16] Collins O, Okeke J. Analysis and control measures for Lassa fever model under socio-economic conditions. In: Journal of Physics: Conference Series. vol. 1734. IOP Publishing; 2021. p. 012049.
[17] Onah IS, Collins OC, Madueme PGU, Mbah GCE. Dynamical system analysis and optimal control measures of Lassa fever disease model. International Journal of Mathematics and Mathematical Sciences. 2020;2020:1–18.
[18] Coetzee C. The breeding season of the multimammate mouse Praomys (Mastomys) natalensis (A. Smith) in the Transvaal Highveld. African Zoology. 1965;1(1):29–39.
[19] Fichet-Calvet E, Lecompte E, Koivogui L, Daffis S, Meulen JT. Reproductive characteristics of Mastomys natalensis and Lassa virus prevalence in Guinea, West Africa. Vector-Borne and Zoonotic Diseases. 2008;8(1):41–48.
[20] Leirs H, Verheyen W, Michiels M, Verhagen R, Stuyck J. The relation between rainfall and the breeding season of Mastomys natalensis (Smith, 1834) in Morogoro, Tanzania. In: Annales de la soci ́et ́e Royale Zoologique de Belgique. vol. 119; 1989. p. 59–64.
[21] Leirs H, Verheyen W, Verhagen R. Spatial patterns in Mastomys natalensis in Tanzania (Rodentia, Muridae). 1996; p 545-556.
[22] Leirs H, Verhagen R, Verheyen W. The basis of reproductive seasonally in Mastomys rats (Rodentia: Muridae) in Tanzania. Journal of Tropical Ecology. 1994;10(1):55–66.
[23] Makundi RH, Massawe AW, Mulungu LS. Reproduction and population dynamics of Mastomys natalensis Smith, 1834 in an agricultural landscape in the Western Usambara Mountains, Tanzania. Integrative Zoology. 2007;2(4):233–238.
[24] Sluydts V, Davis S, Mercelis S, Leirs H. Comparison of multimammate mouse (Mastomys natalensis) demography in monoculture and mosaic agricultural habitat: Implications for pest management. Crop Protection. 2009;28(8):647–654.
[25] Massawe AW, Mulungu LS, Makundi RH, Dlamini N, Eiseb SJ, Kirsten F, et al. Spatial and temporal population dynamics of rodents in three geographically different regions in Africa: implication for ecologically-based rodent management. African Zoology. 2011;46(2):393–405.
[26] Mari ̈en J, Kourouma F, Magassouba N, Leirs H, Fichet-Calvet E. Movement patterns of small rodents in Lassa fever-endemic villages in Guinea. EcoHealth. 2018;15:348–359.
[27] Monadjem A, Themb’a AM, Dlamini N, Eiseb SJ, Belmain SR, Mulungu LS, et al. Impact of crop cycle on movement patterns of pest rodent species between fields and houses in Africa. Wildlife Research. 2011;38(7):603–609.
[28] Ter Meulen J, Lukashevich I, Sidibe K, Inapogui A, Marx M, Dorlemann A, et al. Hunting of peridomestic rodents and consumption of their meat as possible risk factors for rodent-to-human transmission of Lassa virus in the Republic of Guinea. American Journal of Tropical Medicine and Hygiene. 1996;55:661–666.
[29] Tomori O, Fabiyi A, Sorungbe A, Smith A, McCormick JB. Viral hemorrhagic fever antibodies in Nigerian populations. The American journal of tropical medicine and hygiene. 1988;38(2):407–410.
[30] Tobin EA, Asogun D, Akpede N, Adomeh D, Odia I, Gunther S, et al. Lassa fever in Nigeria: Insights into seroprevalence and risk factors in rural Edo State: A pilot study. Journal of Medicine in the Tropics. 2015;17(2):51.
[31] Musa SS, Zhao S, Abdullahi ZU, Habib AG, He D. COVID-19 and Lassa fever in Nigeria: A deadly alliance? International Journal of Infectious Diseases. 2022;117:45–47.
